# 

**Published:** 1887-01

**Authors:** 


### CONTENTS.

COMMUNICATIONS.

Corydon L. Ford, L.L.D., M.D., D.D.S. .... 5
Operative Dentistry .... 8
Tin and Gold Combined as a Filling Material .... 16
A Method of Preparing Dental Tissues for Microscopical Examinations .... 19

SELECTIONS.

Dentistry Not a Specialty of Medicine .... 21
Is Dentistry a Specialty of Medicine? .... 24
Compound Fracture of the Superior Maxillary Bone .... 30
Some Disorders of Adolescence .... 32
Microbes .... 36
Two Cases of Reunion of Cut-off Fingers .... 37
Force of Growing Plants .... 37
Sir Joseph Lister's Operations .... 38
The Importance of Cleanliness .... 38
Prophylaxis more Important than Remedial Treatment .... 39
Left-Handedness .... 44
Surface Filth as a Medium of Disease .... 45
Apparent Recovery from Phthesis .... 46
SALMAGUNDI .... 47
CORRESPONDENCE .... 53

EDITORIAL.

Obituary .... 54-56
Louisiana State Dental Association .... 56

